# Peculiar Case of Brain Abscess Caused by Propionibacterium acnes in an Immunocompetent Individual Without Prior Neurosurgical Intervention: A Case Report and Literature Review

**DOI:** 10.7759/cureus.43647

**Published:** 2023-08-17

**Authors:** Muhammad Tahir, Mary Vansant, Osama Elkadi

**Affiliations:** 1 Pathology and Laboratory Medicine, University of South Alabama Health Hospital, Mobile, USA; 2 Internal Medicine, Navy Medical Center San Diego, San Diego, USA

**Keywords:** propionibacterium, brain abscess excision, brain infection, propionibacterium acnes, anaerobic brain abscess

## Abstract

Propionibacterium acnes (P. acnes) is a slow-growing, anaerobic, gram-positive bacillus that commonly colonizes the skin and is a rare cause of CNS infections. It was previously viewed as a culture contaminant but is now recognized to infrequently cause indolent cases of CNS infections. It is even more rarely associated with abscesses in patients without a prior history of neurosurgical intervention. Due to being a slow-growing bacteria, P. acnes is frequently discovered to be the causative organism after 16S rRNA sequencing. In this case, the culture was positive. There are only five other reported cases of patients with a P. acnes abscess without prior neurosurgical intervention. Here we present the sixth case of an immunocompetent young male who was found to have a P. acnes brain abscess.

## Introduction

Inflammation of the brain can be due to infectious or noninfectious causes. Among infectious etiologies, the most common route of infection is through direct or indirect invasion of the blood-brain barrier. Infection and inflammation of the brain parenchyma can also lead to abscess formation. The rate of uncomplicated recovery for patients with brain abscesses has increased from 33% to 70% over the past five decades [1,2]. Despite such a successful recovery rate, the mortality and morbidity of brain abscess patients are very high, and clinical optimization through quick and accurate diagnosis in a timely manner with proper therapeutic intervention is crucial.

The most common clinical presentation of patients with brain abscesses is a triad of fever, headache, and focal neurological symptoms. Most importantly, the focal neurological deficit can be the sole presentation of a neurological disorder coexisting with a brain abscess. Approximately 86% of patients with abscesses have predisposing conditions such as sinusitis, otitis media, mastoiditis, and meningitis. Neurological surgical interventions are the main cause of brain abscesses, but brain abscesses can also rarely develop in patients without any pre-existing risk factors [3,4].

In immunocompetent individuals, approximately 95% of brain abscess cases are usually polymicrobial and bacterial in origin. The most commonly identified bacterial microorganisms are streptococci (i.e., S. mitis, S. mutans, and S. salivarius), staphylococci (i.e., S. aureus), anaerobes (i.e., actinomyces, Bacteroides), and Enterobacteriaceae. Empirical antibiotic treatment with third-generation cephalosporins and metronidazole is the standard of care. Neurosurgical interventions in the form of abscess aspiration and placement of an external ventricular drain, collection of biopsy fluid, and biopsy of brain parenchyma for diagnostic analysis are helpful diagnostic and therapeutic interventions [5].

## Case presentation

A 21-year-old African American male with no significant past medical history (except migraine headaches) was transferred to the University of South Alabama Health Hospital emergency department with a chief complaint of left-sided weakness. The patient reported that he had been hit in the head two weeks ago with a wooden stick, was knocked off balance, and started having some headaches and left-sided weakness. On physical examination, the patient denies blurry vision, headache, nausea, vomiting, chest pain, diaphoresis, or confusion. Emergency laboratory evaluation revealed an elevated WBC count; otherwise, the remaining labs were unremarkable. Workups for sexually transmitted diseases (STDs), including HIV, rapid plasma reagin (RPR), and hepatitis, were negative. Neurological examination revealed 4/5 grip strength (grips) in the left upper extremities, 5/5 grip strength (grips) in the right upper extremities, 2/5 strength in the left lower extremity, and 5/5 strength in the right lower extremity.

**Figure 1:**
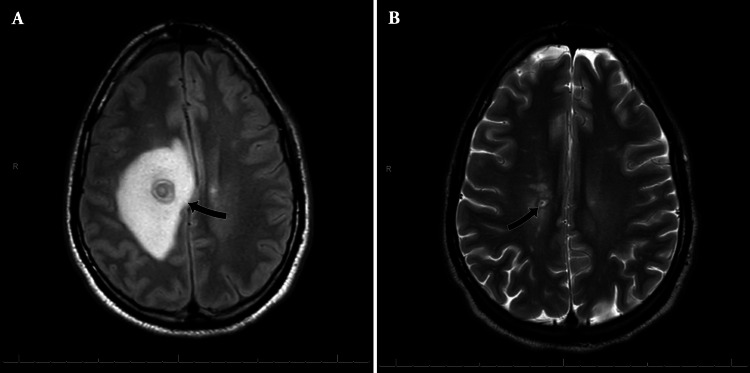
A) MRI with intravenous gadolinium showed a lesion with significant vasogenic edema and midline shift; B) Post-craniotomy MRI showing residual lesion.

A CT of the head without contrast revealed a right periventricular white matter mass measuring 1.2 cm in greatest dimensions. The mass was peripherally hyperdense with an extensive amount of surrounding vasogenic edema, most suggestive of an intracranial abscess. MRI of the brain with intravenous gadolinium revealed a peripherally ring-enhancing lesion in the right corona radiata adjacent to the cingulate gyrus measuring 1.6 x 2.1 cm with continuous ring enhancement. The enhancing portion of the mass measured approximately 0.4 cm. There is significant T2/fluid attenuated inversion recovery (FLAIR) hyperintensity representing edema, which extended into the right frontal, temporal, and parietal white matter. Edema also crossed the midline via the corpus callosum. Edema also crossed the midline via the corpus callosum with a stable 0.8 cm leftward midline shift (Figure 1).

**Figure 2:**
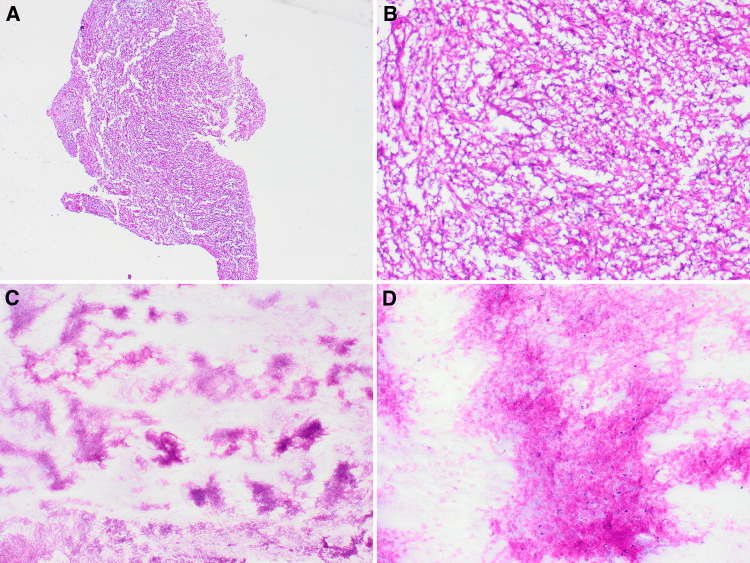
Low-power view showing minute fragments of necrotic debris and a few atypical glial cells (A, 4x; B, 10x). High-power view showing predominantly necrotic debris (C, 20x; D, 40x).

The patient was admitted to the neurosurgical intensive care unit (NSICU), and neurosurgery was consulted. A right burr hole craniotomy for biopsy with stealth navigation was performed. Pathology department was consulted, and a frozen section was performed for intraoperative diagnosis to confirm malignancy versus abscess formation. Evaluation of the frozen section revealed minute fragments of predominantly necrotic tissue with a few atypical glial cells (Figure 2). No definitive malignancy was identified in the frozen section specimen, and more tissue was requested for permanent sections for further work-up.

**Figure 3:**
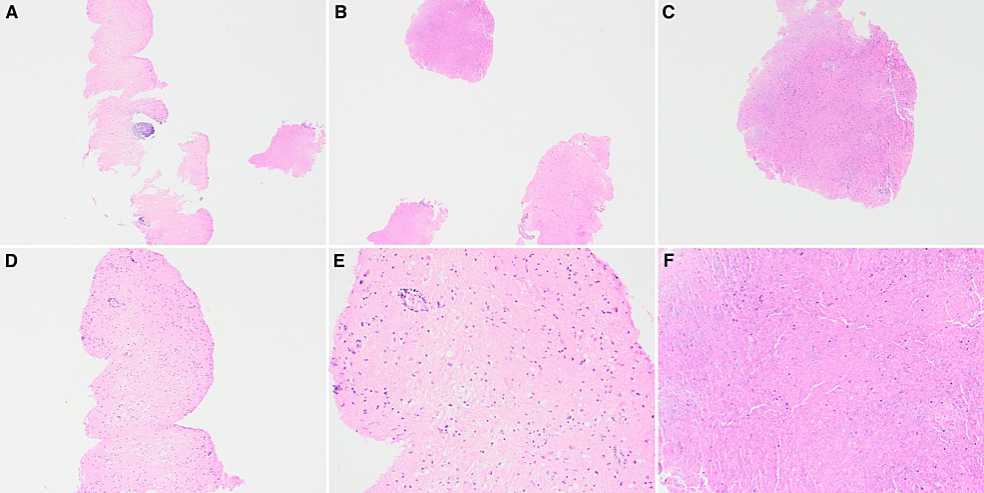
Low-power view showing multiple fragments of necrotic brain parenchyma (A, B, C 4x). Medium and high-power showing fragments of necrotic tissue with macrophages and perivascular inflammatory infiltrate (D, 10x; E, 20x; F, 40x).

Histologic examination of permanent sections revealed fragments of benign brain parenchyma with gliosis, macrophages, and a focal dense perivascular inflammatory infiltrate. Abundant amounts of necrotic tissue were also identified, and multiple immunohistochemistry stains were ordered to rule out an infectious etiology (Figure 3).

**Figure 4:**
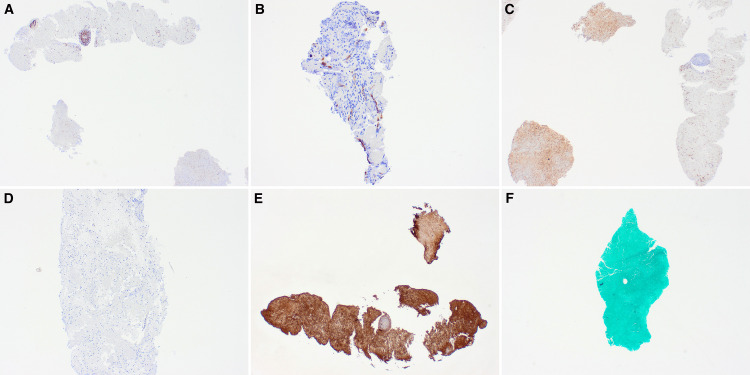
CD3 highlights scattered T lymphocytes (A, 4x). CD34 is focally positive in endothelial cells (B, 4x). CD68 is diffusely positive and highlights the macrophages (C, 4x). The CMV stain is negative (D, 4x). GFAP is diffusely positive, showing reactive gliosis (E, 4x). GMS is negative for microorganisms (F, 4x). CMV: cytomegalovirus; GFAP: glial fibrillary acidic protein; GMS: Grocott methenamine silver

**Figure 5:**
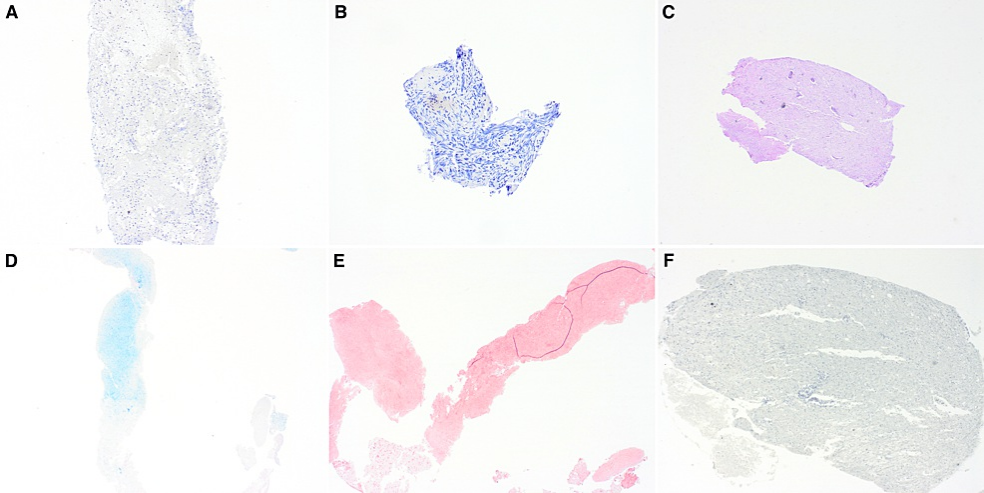
Negative HSV and Ki-67 (A, B, 4x). PASH+D is negative for fungal-like organisms (C, 4x). Negative AFB for Mycobacterium tuberculosis (D, 4x). Gram stain negative for bacteria (E, 4x). Negative Toxoplasma immunostaining (F, 4x). HSV: herpes simplex virus; PAS: periodic acid-Schiff; PAS-D: periodic acid-Schiff with diastase; AFB: acid-fast bacilli

Immunohistochemistry (IHC) was performed to rule out infectious etiologies and malignancies, and highlight the normal brain parenchyma. Immunoperoxidase stains using antibodies against glial fibrillary acidic protein (GFAP), CD68, CD34, CD3, herpes simplex virus (HSV), periodic acid-Schiff (PAS) with diastase (PASH+D), toxoplasma, acid fast bacilli (AFB), Gram stain, Grocott methenamine silver (GMS), cytomegalovirus (CMV), and Ki-67 were performed on representative sections of formalin-fixed paraffin-embedded (FFPE) blocks (Figures 4-5). GFAP showed gliosis, CD68 was positive in macrophages, and CD3 highlighted T lymphocytes. The results support the diagnostic interpretation, and the controls were satisfactory.

**Figure 6:**
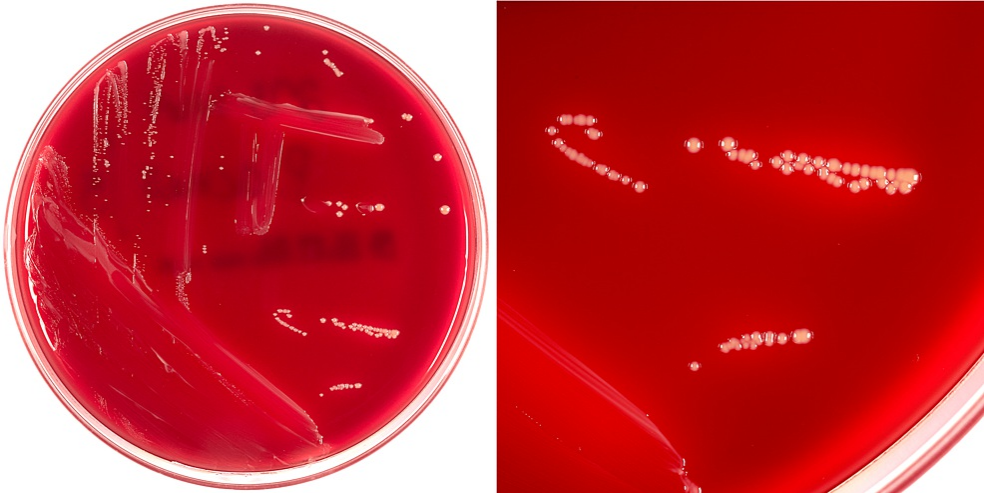
Culture media showing growth of Propionibacterium acnes.

The specimen was sent to microbiology for culture and identification; the culture was positive for Propionibacterium acne ( P. acnes) (Figure 6).

## Discussion

Our patient was hospitalized with left upper extremity and left lower extremity hemiparesis, incoordination, and a headache. A CT scan revealed a 1.2 x 1 cm lesion with vasogenic edema in the right corona radiata, with spectroscopy findings concerning glioblastoma multiforme. The patient underwent a burr-hole craniotomy for a biopsy. Tissue culture was positive for P. acnes after five days. An attempt was made to have 16S rRNA sequencing on the specimen, but the specimen was determined to be inadequate. We do not believe this influenced our conclusion because the slow growth of the organism was consistent with P. acnes. Despite the fact that P. acnes is normal skin flora and a potential contaminant, the sequencing may have provided proof of the infection. The slow growth does not imply contamination or poor collection, as it might with other organisms.

In our literature review, we looked at other cases of P. acnes causing a CNS infection in patients without immunosuppression or prior neurosurgical intervention. In Table 1, we summarize the five cases of brain abscesses caused by P. acnes reported in the literature [5-9]. Most of the case reports found by our initial literature review were consistent with patients who had prior neurosurgical interventions. One study that characterized the microbes mostly involved in post-neurosurgical intracranial infections found P. acnes to be the second most commonly isolated organism after Staphylococcus aureus, at 28.6% [10]. The incidence of P. acnes CNS infections in patients without prior neurosurgical intervention has yet to be described due to its rarity. The mechanism by which the infection occurs after neurosurgery is thought to be direct infection, as P. acnes is part of normal skin flora.

**Table 1:** List of brain abscess cases by Propionibacterium acnes in immunocompetent individuals without prior neurosurgical intervention.

Author	Sex	Age (Years)	Immunocompromised	Other Risk Factors	Site of Infection	Diagnostic Method	Isolated Microbes	
Smith et al., 2022 [5]	M	55	No	None	Brain abscess	Surgical biopsy and culture	P. acnes, cladosporium, nonaeruginosa pseudonomas	
Ramos et al., 1995 [6]	F	28	No	None	Brain abscess	Surgical biopsy and culture	P acnes, s. anguinosus	
Kim et al., 2009 [7]	M	23	No	chronic subdural hematoma	Subdural empyema	Surgical biopsy and culture	P. acnes	
Odunukan et al., 2016 [8]	M	49	No	None	Brain abscess	Surgical biopsy and culture	P. acnes	
Zaffiri et al., 2013 [9]	M	79	No	None	Anterior right frontal lobe	Maxillary sinus culture	P. acnes	

A case report by Odunkan et al. describes that the mechanism for the translocation of P. acnes across the blood-brain barrier is unknown [8]. The risk factors were also not identified in most cases that were reviewed during the literature search. In one patient, they hypothesized a possible risk factor for CNS infection by P. acnes to be plaque psoriasis, which increases the risk of bacteremia. They also discussed the possibility of dental abscesses over time causing a CNS infection by overwhelming the blood-brain barrier [11]. Neither of these risk factors were present in our patient or in the other case report that we reviewed.

Kim et al. reported a case of a subdural empyema with P. acnes as the causative organism [7]. They suspected that due to the patient’s medical history, they may have had a chronic subdural hematoma, which could have been a predisposing factor to the development of the empyema, although it could not be confirmed. The patient also had a history of a head injury a month prior. Our patient also had a history of trauma to the head a couple weeks before presentation. Though we cannot be certain that head trauma contributed to the P. acnes infection in our patient, it is worth considering that head trauma can be a possible risk factor for the unexplained CNS infection.

Viewing the demographic characteristics of the five cases of CNS abscess caused by P. acnes, two cases involved young adult patients. The patient we present is also a young adult, making up half of the reported cases of P. acnes in young adults with the characteristics we describe of being immunocompetent and without prior neurosurgical intervention. It raises the question of whether there is a risk factor in these young adult patients that has yet to be identified.

There are many questions to be raised in this case and the other reported cases involving immunocompetent hosts without prior neurosurgical intervention. Are there other risk factors not being considered in these patients? Are there cases being missed due to the slow growth of P. acnes? The possibility of missed cases is supported by the slow growth of the organism and its high susceptibility to penicillin and cephalosporins, which decrease the need for further investigation in some patients. What is the method of transmission in immunocompetent patients? In immunocompromised patients, it could be suggested that the transmission could be through a break in the skin, as P. acnes is part of the normal skin flora. In immunocompetent individuals, the etiology of transmission remains unclear.

## Conclusions

Intracranial infections by P. acnes are not frequent, but this agent should be considered a potential cause of post-surgical brain abscesses. We emphasize the need to send tissue samples for culture and proper identification of the causative microorganism before giving negative results. We also emphasize the relevance of the Gram stain as a rapid method to guide the diagnosis; the microbiology laboratory must have an adequate methodology for the recovery of this type of microorganism.
